# Species differences and molecular determinant of TRPA1 cold sensitivity

**DOI:** 10.1038/ncomms3501

**Published:** 2013-09-27

**Authors:** Jun Chen, Dawon Kang, Jing Xu, Marc Lake, James O. Hogan, Chaohong Sun, Karl Walter, Betty Yao, Donghee Kim

**Affiliations:** 1 Neuroscience and Early Discovery, Building AP9-1125, North Waukegan Road, North Chicago, Illinois 60064-6125, USA; 2 Department of Physiology and Biophysics, The Chicago Medical School, Rosalind Franklin University of Medicine and Science, 3333 Green Bay Road, North Chicago, Illinois 60064, USA; 3 Department of Physiology, Medical Research Centre for Neural Dysfunction, Institute of Health Sciences, Gyeongsang National University School of Medicine, Jinju 660-751, Korea; 4 Global Protein Science, Research and Development, AbbVie, Inc., Building AP9-1125, North Waukegan Road, North Chicago, Illinois 60064-6125, USA

## Abstract

TRPA1 is an ion channel and has been proposed as a thermosensor across species. In invertebrate and ancestral vertebrates such as fly, mosquito, frog, lizard and snakes, TRPA1 serves as a heat receptor, a sensory input utilized for heat avoidance or infrared detection. However, in mammals, whether TRPA1 is a receptor for noxious cold is highly controversial, as channel activation by cold was observed by some groups but disputed by others. Here we attribute the discrepancy to species differences. We show that cold activates rat and mouse TRPA1 but not human or rhesus monkey TRPA1. At the molecular level, a single residue within the S5 transmembrane domain (G878 in rodent but V875 in primate) accounts for the observed difference in cold sensitivity. This residue difference also underlies the species-specific effects of menthol. Together, our findings identify the species-specific cold activation of TRPA1 and reveal a molecular determinant of cold-sensitive gating.

## Abstract (web summary)

TRPA1 ion channels act as thermosensors across different species; however, studies on their role in noxious cold sensation have provided conflicting results in mammals. Chen *et al*. show that these discrepancies arise because cold activates rat and mouse TRPA1 but not human or rhesus monkey TRPA1.

## Graphical abstract

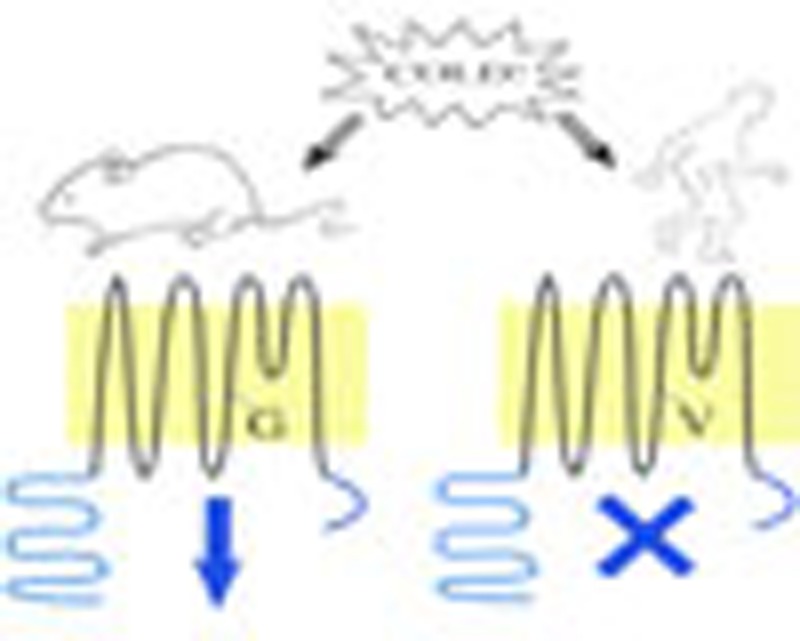

As a member of the transient receptor potential (TRP) ion channel family, TRPA1 functions as a sensor for noxious and electrophilic compounds, including pungent natural products (for example, allyl isothiocyanate or AITC) and environmental irritants (for example, acrolein)^1^,^2^,^3^. TRPA1 can also be activated by a plethora of endogenous molecules involved in pain, inflammation and oxidative stress (for example, 4-hydroxynonenal and H_2_O_2_)^4^,^5^. Recently, a gain of function mutation of TRPA1 was linked to familial episodic pain syndrome in humans^6^. These findings, combined with mounting preclinical evidence^2^,^7^,^8^,^9^,^10^, have established TRPA1 as a promising drug target for pain, inflammation and respiratory diseases.

Despite the recent progress, a fundamental question remains as to whether TRPA1 is a receptor for noxious cold^11^,^12^. Cold activation of TRPA1 was first observed in Chinese Hamster Ovary cells expressing mouse TRPA1 (ref. 8), wherein noxious cold elicited Ca^2+^ influx and non-selective cation currents with a temperature threshold of 17 °C. Similar results were reported in subsequent studies^6^,^13^,^14^,^15^. In contrast, several other studies found that cold did not activate TRPA1 (refs 3, 16, 17). The underlying cause of the discrepancy is unknown, and a direct comparison of these results is confounded by the use of different experimental conditions and the use of clones from various species. Behaviour studies using knockout mice were expected to settle the controversy; however, they only yielded more conflicting results, possibly because of differences in host strain and behaviour end points^2^,^7^,^14^,^18^. Interestingly, TRPA1 from several invertebrate and ancestral vertebrate species (for example, fly, mosquito, frog, lizard and snake) is heat-sensitive instead of being cold-sensitive^19^,^20^,^21^,^22^,^23^. Additionally, we and others observed that rodent and human TRPA1 responded differently to chemical ligands^24^,^25^,^26^, prompting us to investigate TRPA1 cold sensitivity across different species.

In the current study, we systematically analysed the cold sensitivity of TRPA1 from the following four mammalian species: mouse, rat, human and rhesus monkey. Using intracellular Ca^2+^ assay, whole-cell recordings and single-channel recordings, we show that cold activates TRPA1 of the two rodent species but not the two primate species. Through testing chimeric and mutant channels, we identify a single residue that is necessary for the cold activation of rodent TRPA1. In addition to revealing the molecular determinants of cold-sensitive gating, our findings expand the evolutionary divergence of TRPA1.

## Results

### Cold activates rodent but not primate TRPA1

**Figure 1:**
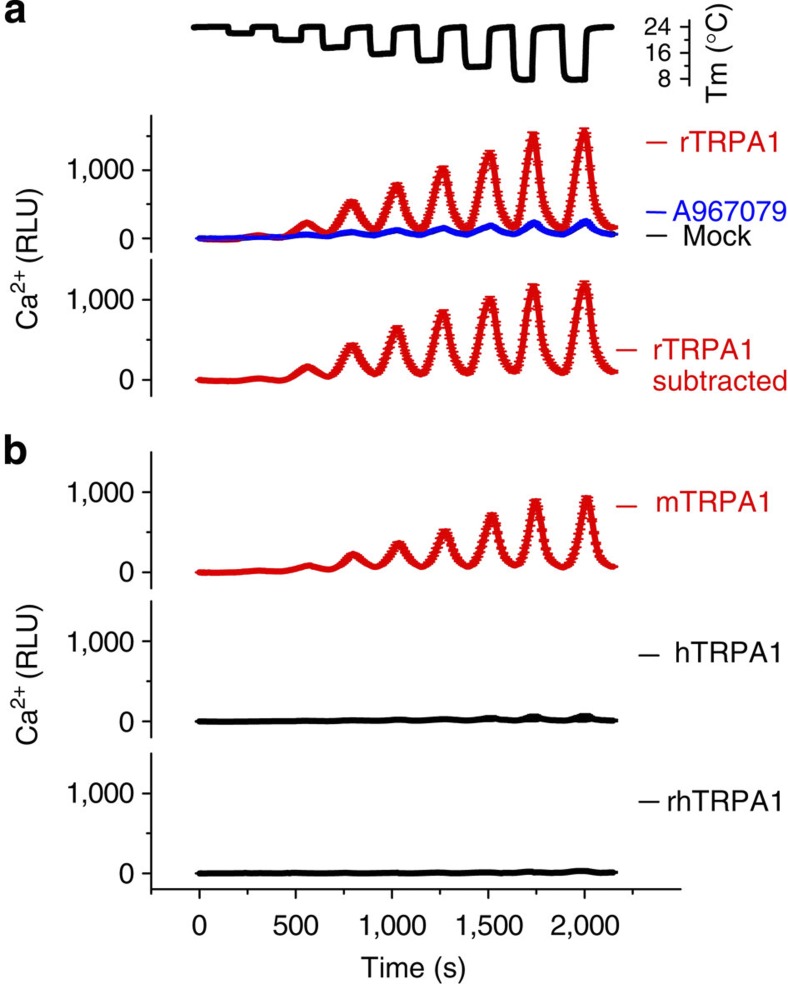
Cold responses of TRPA1 species variants in Ca^2+^ assay. (**a**) In rTRPA1-expressing cells, lowering temperature in steps (from 24 to 20, 18, 16, 14, 12, 10 and 8 °C) evoked progressively larger Ca^2+^ influx, as reflected by increases in relative light unit (RLU). Signals in A967079 (5 μM)-treated rTRPA1 cells and mock-transfected cells were small and overlapped. A967079-specific signals (rTRPA1 subtracted) were obtained to represent TRPA1 activities. (**b**) Cold increased (Ca^2+^) in cells expressing mTRPA1 but not hTRPA1 or rhTRPA1. Fluorescence traces are shown as mean±s.d. from 12 wells. More than five transfections were performed.

Rat (r-), mouse (m-), human (h-) and rhesus monkey (rh-) TRPA1 were transiently expressed in HEK293-F cells and tested in a FLIPR-based Ca^2+^ assay (Supplementary Fig. S1). The maximal responses to the electrophilic agonist AITC were similar among four channel types, indicating comparable levels of expression and activities. Next, we tested the cold sensitivity of the four channels side-by-side, using a device capable of controlling temperature precisely across a 384-well plate^27^. Cooling progressively increased intracellular [Ca^2+^] in rTRPA1-expressing cells, whereas relatively tiny signals were elicited in mock-transfected cells, or when rTRPA1-expressing cells were treated with 5 μM of A967079, a TRPA1-specific antagonist (Fig. 1a)^28^. The threshold of rTRPA1 cold activation, when defined as a 20% increase in A967079-specific signals, was ~18 °C. The increase in intracellular [Ca^2+^] in response to cold was reversible and independent of holding temperature, as difference in holding temperatures (24 or 32 °C) did not significantly affect cold responses (Supplementary Fig. S2). Similar to rTRPA1, mTRPA1 was activated by cold with a temperature threshold of 16 °C (Fig. 1b). In contrast, under the identical condition, cold did not activate hTRPA1 or rhTRPA1 (Fig. 1b), suggesting a species difference in cold sensitivity.

**Figure 2:**
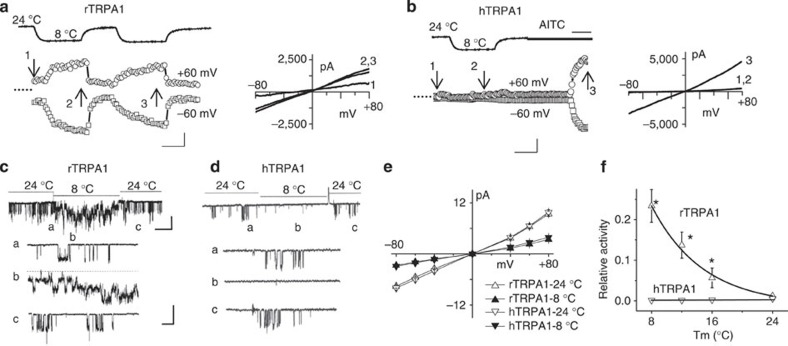
Cold sensitivity of rTRPA1 and hTRPA1 in whole-cell and single-channel recordings. (**a**) Time course and current traces of rTRPA1 whole-cell currents. Numbers indicate time points and dotted line indicates zero current level. Scale bars are 1 nA and 50 s. (**b**) hTRPA1 whole-cell currents in response to cold and AITC (100 μM). Scale bars are 2 nA and 50 s. (**c**,**d**) Cell-attached single-channel recordings of r- and hTRPA1 at 24 and 8 °C (−60 mV). Expanded current tracings are shown as indicated. Scale bars are 5 pA and 100 ms. (**e**) Current–voltage relationships of rTRPA1 and hTRPA1 at 24 and 8 °C (−60 mV). AITC (100 μM)-evoked currents were used for hTRPA1 due to lack of cold activation. *n*=4–5. (**f**) Relative channel activity as function of temperature. *n*=4–5. Channel activity in response to cold was determined by normalizing *NP*o of cold response to *NP*o of AITC response. rTRPA1 activities were fitted with an exponential regression*. n*=5. *P<0.05 (Student’s *t-*test). All error bars are s.d.

The effects of cold on r- and hTRPA1 were further tested in whole-cell recordings. To minimize Ca^2+^-mediated effects^17^, K^+^/Na^+^-based, nominally Ca^2+^-free intracellular and extracellular solutions were used. Cells were held at 0 mV and currents were elicited by a 500-ms voltage ramp (−80 to +80 mV) applied every 3 s (Fig. 2a). In rTRPA1-expressing cells, cold (8 °C) elicited large inward and outward currents were fully reversible and reproducible in the same cell. In hTRPA1-expressing cells, cold (8 °C) failed to elicit currents despite the strong activation of TRPA1 by AITC (100 μM) (Fig. 2b).

### Cold increases single-channel opening of rodent TRPA1

To determine cold-induced changes in channel kinetics, cell-attached single-channel recordings were performed. In Ca^2+^-free solutions, cooling produced a marked increase in single-channel activity of rTRPA1 by eliciting long-lasting openings (Fig. 2c). The single-channel currents were blocked by A967079 (Supplementary Fig. S3). In contrast, cooling reduced the basal activity of hTRPA1 (Fig. 2d). Similar results were obtained in external solutions containing 1 mM Ca^2+^; therefore, the difference in cold responses was not dependent on external Ca^2+^ (Supplementary Fig. S4).

Gradual lowering of the temperature from 24 to 8 °C progressively reduced the amplitudes of single-channel currents (Supplementary Fig. S5), prompting us to further examine the temperature effects on channel conductance. The current–voltage relationships for r- and hTRPA1 were superimposable at either 24 or 8 °C, and unitary conductance at 8 °C was reduced for both channels (Fig. 2e). For example, for rTRPA1, the unitary conductance at −40 mV was 92.5±4.9 pS at 24 °C and 34.5±2.5 pS at 8 °C (*n*=4–5); for hTRPA1, the unitary conductance was 87.3±3.9 pS at 24 °C and 30.3±2.2 pS at 8 °C (*n*=4–5). The reduction in conductance was maintained in a K^+^-based solution (Supplementary Fig. S6). Despite similar decreases in channel conductance, cold reduced the basal opening of hTRPA1 but increased the opening of rTRPA1 (Fig. 2f).

**Figure 3:**
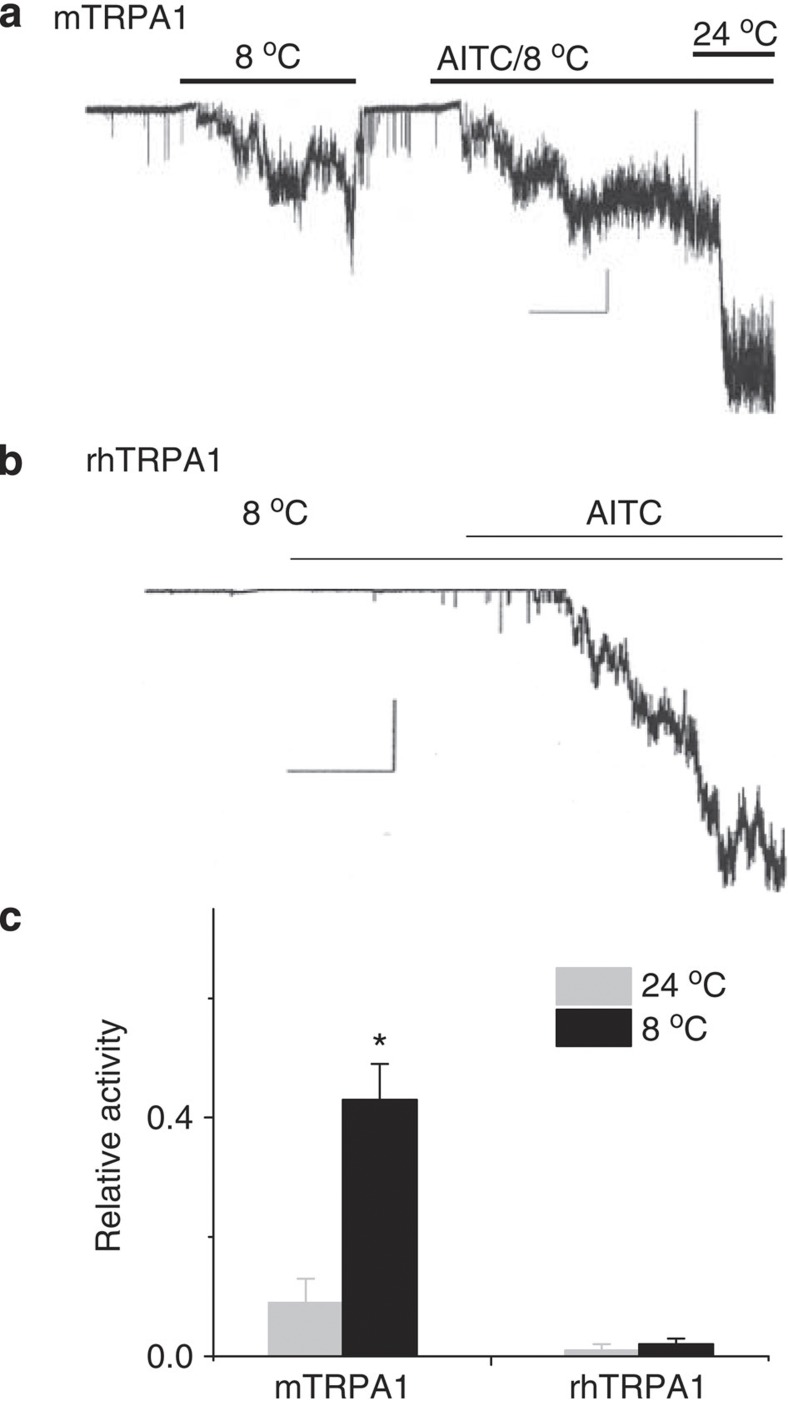
Cold activates mTRPA1 but not rhTRPA1. (**a**,**b**) Representative m- and rhTRPA1 current traces. Scale bars are 5 pA and 20 s for mTRPA1, and 5 pA and 30 s for rhTRPA1. (**c**) Relative activity of m- and rhTRPA1 at 24 and 8 °C. Relative activity was determined by normalizing *NP*o of cold response to *NP*o of AITC response. *n*=6 for mTRPA1 and *n*=5 for rhTRPA1. *P<0.05 from Student’s *t*-test. Error bars are s.d.

The cold sensitivities of mTRPA1 and rhTRPA1 were also examined in cell-attached single-channel recordings. Cold increased opening of mTRPA1 (Fig. 3a,c), in agreement with previous reports^14^,^15^, but failed to affect rhTRPA1 (Fig. 3b,c). Collectively, data from Ca^2+^ assay, whole-cell and single-channel recordings demonstrate that cold activates two rodent but not two primate TRPA1 channels.

### S5 segment and S5-S6 linker determine cold sensitivity

**Figure 4:**
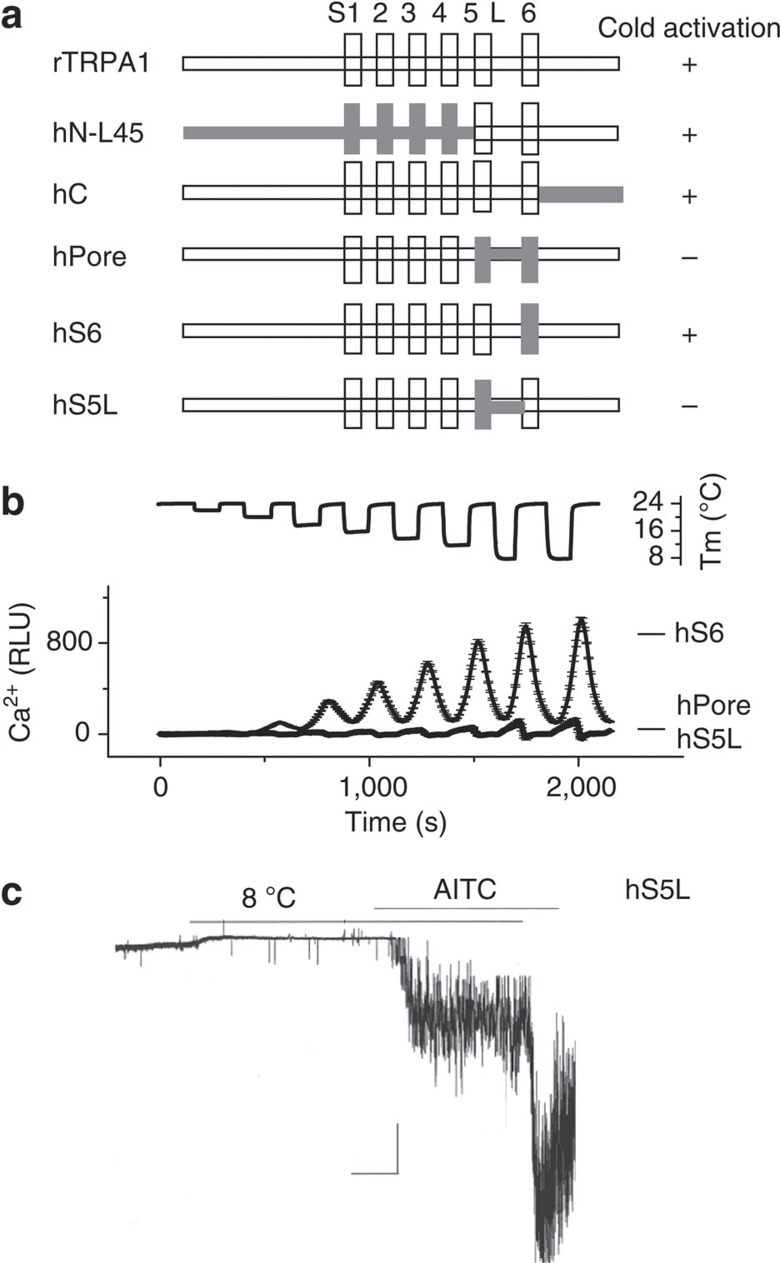
S5 and S5-S6 linker domains are required for cold activation of rTRPA1. (**a**) Schematic representation of chimeras. The amino-acid compositions of chimeras are described in Methods. (**b**) Cold-activated hS6 but not hPore or hS5L in the Ca^2+^ assays*. n*=12. Five independent transfections were tested. (**c**) Representative traces of cell-attached single-channel recording of hS5L at 24 and 8 °C and then with AITC (100 μM). Note the changing temperature from 8 to 24 °C increased amplitudes of AITC currents, indicating that the conductance sensitivity to cold remains intact. Scale bars are 5 pA and 20 s.

How does cold have different effects on rodent and primate channels? The sequence homology is relatively low between rodent and primate TRPA1 (79% identity) but very high within each order (97% identity in full-length and 100% identity in pore domain). To identify the molecular basis of the species difference, we made rTRPA1–hTRPA1 chimeras by systematically introducing various domains of hTRPA1 into rTRPA1 background (Fig. 4a). All chimeras were functional in the Ca^2+^ assay, with AITC (100 μM) response ranging from 85 to 113% of wild-type rTRPA1. Introducing domains from *N*-terminus to L45 linker of hTRPA1 (hNL45), or domain containing *C*-terminus of hTRPA1 (hC), did not affect cold activation (Fig. 4a and Supplementary Fig. S7), whereas introducing the pore domain (hPore, containing S5, S5-S6 linker and S6 from hTRPA1) abolished cold activation (Fig. 4a,b). Within the pore domain, introducing the S6 segment of hTRPA1 (hS6) retained cold activation, with a threshold of 18 °C (Fig. 4b); in contrast, introducing the S5 and S5-S6 linker from hTRPA1 (hS5L) abolished cold activation (Fig. 4b). In single-channel recordings, cold (8 °C) failed to activate hS5L, whereas AITC (100 μM) elicited strong activation (Fig. 4c). Thus, residues critical for cold activation are localized in the S5 and S5-S6 linker domains.

### rTRPA1-G878V abolishes cold sensitivity of rTRPA1

**Figure 5:**
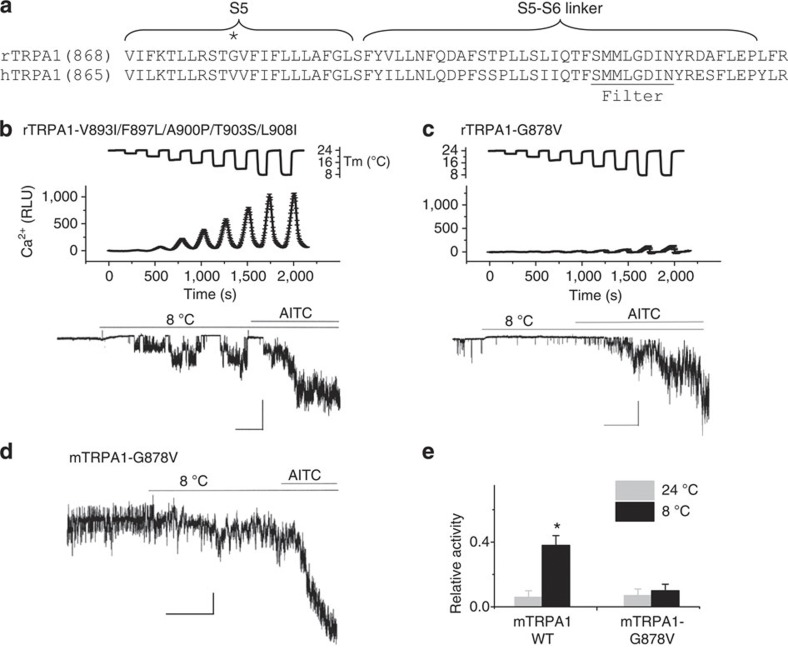
A single residue determines cold sensitivity of r- and mTRPA1. (**a**) Sequence alignment of S5 and S5-S6 linker domains of r-and hTRPA1. The critical residues G878 (rTRPA1) is marked by *. (**b**,**c**) rTRPA1-V893I/F897L/A900P/T903S/L908I but not rTRPA1-G878V retained cold sensitivity in Ca^2+^ assay (*n*=12) and single-channel recordings (*n*=4). Scale bars are 10 pA and 25 s (left) and 10 pA and 50 s (right). (**d**) Representative single-channel recordings of mTRPA1/G878V in responses to cold and AITC (100 μM). Scale bars are 4 pA and 50 s. (**e**) Relative activity of wild type and mTRPA1/G878V, as determined by normalizing *NP*o against AITC response. *n*=5. *P<0.05 from Student’s *t*-test. Error bars are s.d.

There is an 11-residue difference between r- and hTRPA1 in the S5 and S5-S6 linker domains (Fig. 5a). Each of the 11 rTRPA1 residues was substituted with equivalent hTRPA1 residue individually (for example, rTRPA1-F870L) or in combination (for example, rTRPA1-V893I/F897L/A900P/T903S/L908I, rTRPA1D923E/A924S or rTRPA1-L929Y/F930L). All mutations tested, except one, retained cold activation (for example, rTRPA1-V893I/F897L/A900P/T903S/L908I in Fig. 5b). In contrast, a single-residue substitution in the S5 domain, rTRPA1-G878V, abolished cold activation while retaining AITC activation (Fig. 5c). The same mutation in mTRPA1 (that is, mTRPA1-G878V) also abolished cold activation (Fig. 5d,e). Therefore, G878 is critical for cold activation of both rTRPA1 and mTRPA1.

### rTRPA1-G878V confers hTRPA1-like menthol response to rTRPA1

**Figure 6:**
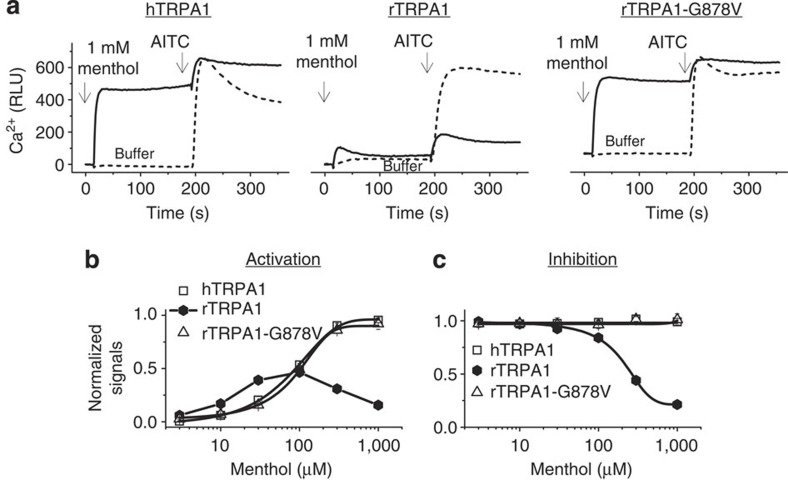
G878 determines species-specific menthol responses. (**a**) In Ca^2+^ assay, 1 mM menthol activated hTRPA1, rTRPA1-G878V but blocked rTRPA1 activation by AITC (30 μM). Solid line: addition of menthol followed by AITC; dotted line: addition of buffer followed by AITC. (**b**) Activation dose–response of menthol as normalized against responses of 30 μM AITC. EC_50_ was 95±4 μM for hTRPA1, and 103±7 μM for rTRPA1-G878V, respectively. Note the bell-shaped activation dose–response of rTRPA1. (**c**) Inhibition dose–responses of menthol. Inhibition of AITC (30 μM)-evoked responses were plotted. IC_50_ of menthol was 245±11 μM on rTRPA1. Menthol did not inhibit hTRPA1 or rTRPA1-G878V. *n*=12, values above are ±s.d.

High concentrations of menthol (for example, 1 mM) activate human TRPA1 and block AITC activation of rodent TRPA1, thereby producing effects that are opposite to those produced by cold^26^,^29^,^30^. Interestingly, G878 of rTRPA1 and its equivalent hTRPA1 residue (V875) were previously found to be responsible for the species-specific effects of menthol, as hTRPA1-V875G conferred menthol blockade to hTRPA1 (ref. 26). Conversely, we found that the reverse mutation (rTRPA1-G878V) abolished menthol blockade and introduced menthol activation (Fig. 6a,b). Therefore, rTRPA1-G878V restores the properties of hTRPA1, including activation by menthol and lack of activation by cold.

## Discussion

Cold sensitivity of TRPA1 has been a controversial issue. Cold activation of TRPA1 was reported by some groups^1^,^8^,^13^,^14^,^15^ but disputed by others^3^,^16^,^17^,^31^. Efforts to reconcile these previous findings were hindered by differences in experimental conditions including the use of TRPA1 from different species (Summarized in Supplementary Table S1). Here we have characterized the cold sensitivity of four mammalian TRPA1 channels under identical experimental conditions. We show that rat and mouse TRPA1s are activated by cold, consistent with several previous studies using m- or rTRPA1 (refs 1, 8, 13, 14, 15). Furthermore, we show that cold increases the open probability of rat and mouse TRPA1 at the single-channel level^14^,^15^. We find that h- and rhTRPA1s are not activated by cold, consistent with several previous studies using human TRPA1 (refs 3, 16, 17).

Although collectively our findings are in line with most of the literature data, and suggest that the discrepancy on TRPA1 cold sensitivity is because of species difference, not all literature data agree with our findings. Nagata *et al*.^31^ mentioned the lack of cold activation of mouse TRPA1; however, they did not report actual data or experimental conditions, preventing a direct comparison with studies from us and others^1^,^8^,^14^,^15^. Bandell *et al*.^1^ and Kremeyer *et al*.^6^ reported cold-evoked, instantaneous whole-cell currents in *Xenopus* oocyte and HEK293 cells expressing human TRPA1. In contrast, Jordt *et al*.^3^, Cordero-Morales *et al*.^16^, Zurborg *et al*.^17^ and we did not observe cold activation of human TRPA1. Although the exact underlying causes are unknown, several factors could contribute to the discrepancy. First, there might be sequence variation of human complementary DNA (cDNA) clones, difference in expression levels and cooling application or other experimental conditions. Second, the cold-activated hTRPA1 currents observed by Bandell *et al*.^1^ and Kremeyer *et al*.^6^ could be mediated by an indirect, Ca^2+^-related mechanism^17^, as Ca^2+^ was used in extracellular recording solutions. Third, cold might activate hTRPA1 but to a much lesser degree (relative to rodent TRPA1), which eluded detection by us and others^3^,^16^,^17^. Compared with previous studies, we employed different methodologies (Ca^2+^ assay, whole-cell and single-channel recordings), tested different ionic conditions (with and without Ca^2+^) and used agonist and antagonist controls (AITC and A967079) throughout the experiments. The side-by-side comparison of fully sequenced TRPA1 from four species, independently performed in two separate laboratories, demonstrate that cold activates rodent but not primate TRPA1. The characterization of dozens of mutant channels and identification of single residue critical for cold activation provide further evidence for the species difference in cold sensitivity.

Our current findings extend the spectrum of TRPA1 function from being heat-sensitive in invertebrates and ancestral vertebrates (that is, fly, mosquito, frog, snake and lizard)^19^,^20^,^21^,^22^, to being cold-sensitive in rodents, and to being thermal-insensitive in primates (Table 1). This is in direct contrast to the electrophilic sensitivity, which is conserved across ~500 million years of animal evolution^32^,^33^. The divergence in thermal sensitivity may be a result of adaptive evolution, as TRPA1 evolves to suit its physiological function in respective species. The evolution of TRPA1 in temperature sensitivity may have been influenced by the emergence of other thermal-sensitive ion channels and receptors^22^. For example, TRPV1 first emerged in ancestral vertebrates, and all TRPV1 channels tested, thus (for example, from frog, snake, chicken, rodents and primates), are heat sensitive^22^,^34^. It will be interesting to determine how these functionally related genes interplay during the course of evolution.

Structural elements involved in heat-sensitive gating have been identified for several TRP channels, including TRPV3 (pore), TRPV1 (pore, *N*-terminus, *C*-terminus), snake TRPA1 and *Drosophila* TRPA1 (*N*-terminus)^16^,^27^,^35^,^36^,^37^,^38^. However, little is known about the molecular basis of cold-sensitive gating. Here we found that a single mutation in the S5 transmembrane domain, G878V, abolishes cold activation of r- and mTRPA1. Interestingly, at this position, the glycine residue (that is, G878) is present only in r- and mTRPA1, whereas a valine (that is, V875) is conserved in the thermo-insensitive h- and rhTRPA1, as well as in the heat-sensitive rattle snake TRPA1 (Supplementary Fig. S8). In our hands, the reverse mutation (hTRPA1-V875G) failed to confer cold sensitivity (that is, no activation of single channels or Ca^2+^ signal), suggesting that G878 is essential but not the only requirement for cold activation. These results are reminiscent of previous findings of several TRPA1 ligands^24^,^25^. Thioaminals and caffeine are activators of rodent TRPA1 and blockers of human TRPA1 (refs 24, 25). rTRPA1-A946S abolished activation of rTRPA1 by thioaminals; however, the reverse mutation could not confer activation of hTRPA1. Similarly, mTRPA1-M268P abolished activation by caffeine, whereas the reverse mutation failed to confer activation by caffeine.

Remarkably, the species-specific effects of cold and menthol are determined by a single-residue difference (rTRPA1-G878 and hTRPA1-V875) (Fig. 6). Cold activates rodent and inhibits primate TRPA1 (Figs 1, 2), whereas menthol (for example, 1 mM) inhibits rodent and activates primate TRPA1 (Fig. 6)^26^,^29^,^30^. Therefore, it is likely that menthol and cold elicit opposite conformational change in each residue (for example, G878 versus V878). How such conformational changes transduce to channel opening or closing is interesting but is difficult to assess without crystallographic structures. As the glycine residue is unique in possessing the smallest side chain, affording high level of flexibility to adjacent residues and having a strong propensity to break α-helical structure, it will be interesting to explore how such properties affect channel gating. Although more work is required, our current data demonstrate that two distinct stimuli converge on the same S5 residues, implying their essential role in channel gating. Interestingly, a recent study of heat-activated *Drosophila* TRPA1 showed that residues within the putative pore region were critical for warmth sensitivity^39^. Although the exact locations of residues critical for cold (S5 domain in rodent TRPA1) and warmth (pore domain in *Drosophila* TRPA1) are different, they are likely located within the same critical region that modulates gating.

Currently, rodents are used as the default species in TRPA1 research and drug discovery^2^,^7^. It is tempting to extrapolate findings from rodents to humans; however, such practice can be intrinsically flawed. For example, in rodents, TRPA1 has a role in cold allodynia and TRPA1 antagonists attenuate cold allodynia under neuropathic conditions^9^,^13^,^28^; however, extending such findings to humans is not warranted because of the lack of cold sensitivity of human TRPA1. Additionally, many human TRPA1 antagonists exhibit species difference towards rodent channels, manifested as reduced potency or switching to become agonists^40^. These compounds, albeit of potential utility in treating human diseases, cannot be advanced through the current drug-development process that heavily relies on rodent models. On a positive note, we recently showed that rhesus monkey and human channels share the same pharmacology profile^29^. Our current findings further suggest that rhesus monkey should serve as a surrogate species for TRPA1 drug development.

## Methods

### Molecular biology and transient expression

Full-length cDNAs for rTRPA1 (NM_207608), mTRPA1 (NM_177781), hTRPA1 (NM_007332) and rhTRPA1 (XP_001083172) were cloned in pcDNA3.1/V5-His Topo vector (Invitrogen). rTRPA1/hTRPA1 chimeras were generated using overlapping PCR. hNL45: hTRPA1(1–856)/rTRPA1(870–1125); hPore: rTRPA1(1–869)/hTRPA1(857–946)/rTRPA1(950–1125); hS5L: rTRPA1(1–869)/hTRPA1(857–932)/rTRPA1 (936–1125); hS6: rTRPA1(1–935)/hTRPA1(933–946)/rTRPA1(950–1125); hC: rTRPA1(1–935)/hTRPA1(933–1119). Individual mutations were introduced using QuikChange Site-directed Mutagenesis Kit (Stratagene). Transient expression in HEK293-F cells was performed using FreeStyle 293 Expression System (Invitrogen). In a typical transfection of 3 × 10^7^ cells (30 ml volume), 30 μg of plasmid DNA and 40 μl 293fectin were used.

### Ca^2+^ assay

Ca^2+^ assays were performed using a Ca^2+^ fluorescence dye (R8033) and FLIPR^TETRA^ system (Molecular Device) in 384-well format, allowing direct comparison of multiple samples within the same plate. Five or more independent transfections were performed for each of the wild-type channels (r-, m-, h- and rhTRPA1); three or more independent transfections were performed for each mutant. All channels were functional and had similar responses to AITC. Temperature stimulation was applied through a device that changed temperature across 384-well plates with homogeneity of <0.5 °C (ref. 27).

### Electrophysiology

All electrophysiological experiments were repeated at least four times, and representative tracings were shown, together with plots of averaged data. AITC responses were obtained at the end of recordings to serve as positive control. Whole-cell and single-channel recordings were performed using a patch clamp amplifier (Axopatch 200, Axon Instruments, Union City, CA). Current was filtered at 3 kHz using an 8-pole Bessel filter (−3 dB; Frequency Devices, Haverhill, MA) and transferred to a computer using the Digidata 1320 interface (Axon Instruments) at a sampling rate of 20 kHz. Borosilicate glass pipettes with tip resistance of ~4 megaohms were used. Unless otherwise noted, a Na^+^/K^+^-based pipette/bath solution was used, containing (in mM): 117 NaCl, 5 KCl, 1 MgCl_2_, 11 glucose and 23 NaHCO_3_ (pH 7.3). CaCl_2_ was added to the solution when desired. A K^+^-based pipette/bath solution that contains (in mM) 150 KCl, 1 MgCl_2_, 10 HEPES and 11 glucose (pH 7.3) was also used for determining unitary conductance. Temperature of perfusion solution was controlled using the Bipolar Temperature Controller and a dual In-line cooler (Warner Instruments). The minimum duration was set at 0.05 ms for single-channel analysis. Current tracings shown in the figures were filtered at 1 kHz. The threshold for detection of channel opening was set at 50%. For analysis of multiple openings, threshold was set at equal multiple levels. *NP*o (where *N* is the number of channels in the patch, and *P*o is the probability of a channel being open) was determined from 30 to 60 s of recording of openings in response to cold or 100 μM AITC (*NP*o). Relative channel activity was determined by normalizing *NP*o of cold response to *NP*o of AITC response.

## Author contributions

J.C. designed the study, made the initial findings, collected and analysed data and wrote the manuscript. D. Kang and J.O.H. performed electrophysiology experiments. J.X., M.L., C.S., K.W. and B.Y. prepared the reagents. D. Kim designed and performed electrophysiology experiments, analysed data and wrote the manuscript. All authors reviewed manuscript drafts, provided input on the content and approved the final version.

## Additional information

**How to cite this article:** Chen, J. *et al*. Species differences and molecular determinants of TRPA1 cold sensitivity. *Nat. Commun.* 4:2501 doi: 10.1038/ncomms3501 (2013).

## Supplementary Material

Supplementary Information Supplementary Figures S1-S8

## Figures and Tables

**Table 1:** Evolutionary divergence in thermal sensitivity and conservation in electrophile sensitivity of TRPA1.

**Species**	**Temperature sensitivity**	**Electrophile sensitivity**	**Identity to hTRPA1 (%)**	**Accession number**
Mosquito	Heat	Yes	35	XP_001843993
Fruit fly (*dTRPA1*)	Heat	Yes	36	CG5751
Rattle snake	Heat	Yes	63	ADD82930
Frog	Heat	Yes	56	AB693190
Lizard	Heat	Yes	63	AB693189
Mouse	Cold	Yes	79	NP_808449
Rat	Cold	Yes	79	NP_997491
rhesus monkey	Insensitive	Yes	97	XP_001083172
Human	Insensitive	Yes	—	NM_0073332

All channels shown above derive from TRPA1 clade, the common ancestors of vertebrate and invertebrates.
