# Supplementary material for: Species differences and molecular determinant of TRPA1 cold sensitivity
Source: Nat Commun. 2013 Sep 27;4:2501. doi: 10.1038/ncomms3501 (PMC3791479; doi:10.1038/ncomms3501)
Supplement: Supplementary Information — Supplementary Figures S1-S8 [file ncomms3501-s1.pdf]

Supplementary Figure S1

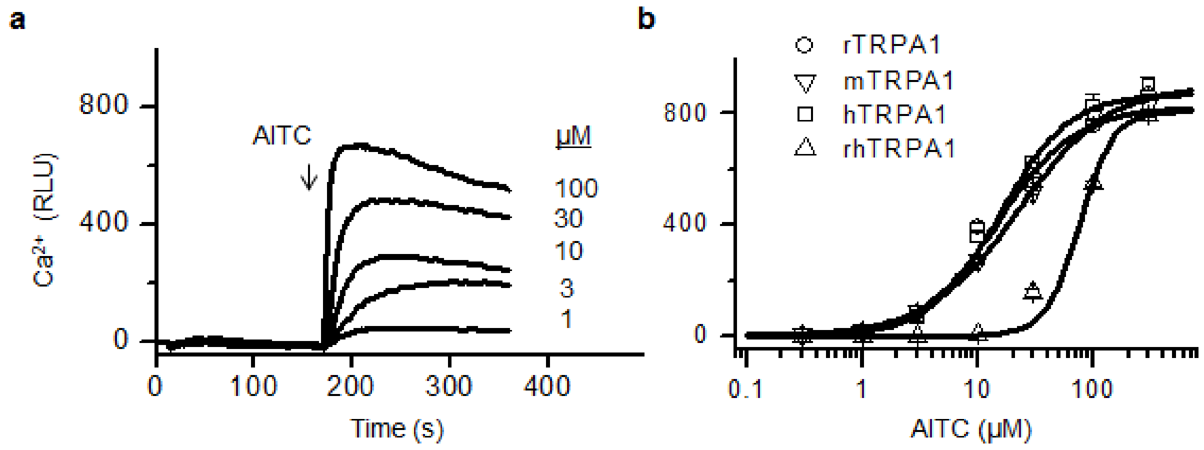

Supplementary Figure S1. AITC responses of TRPA1 species variants in Ca^{2+} assay. **(a)** AITC evoked increase of intracellular Ca2+ in HEK293-F cells expressing rTRPA1 in a concentration-dependent manner, as reflected by increases of relative light units (RLU, Left). **(b)** Concentration dose-responses of AITC activation. EC50 of AITC was 15.3 ± 1.1, 22.2 ± 1.8, 14.9 ± 0.9 and 75.5± 2.7 µM for r-, m-, h- and rh-TRPA1, respectively. Note that the maximal responses were comparable among the four channel types. n = 8. Error bars are s.d.

Supplementary Figure S2

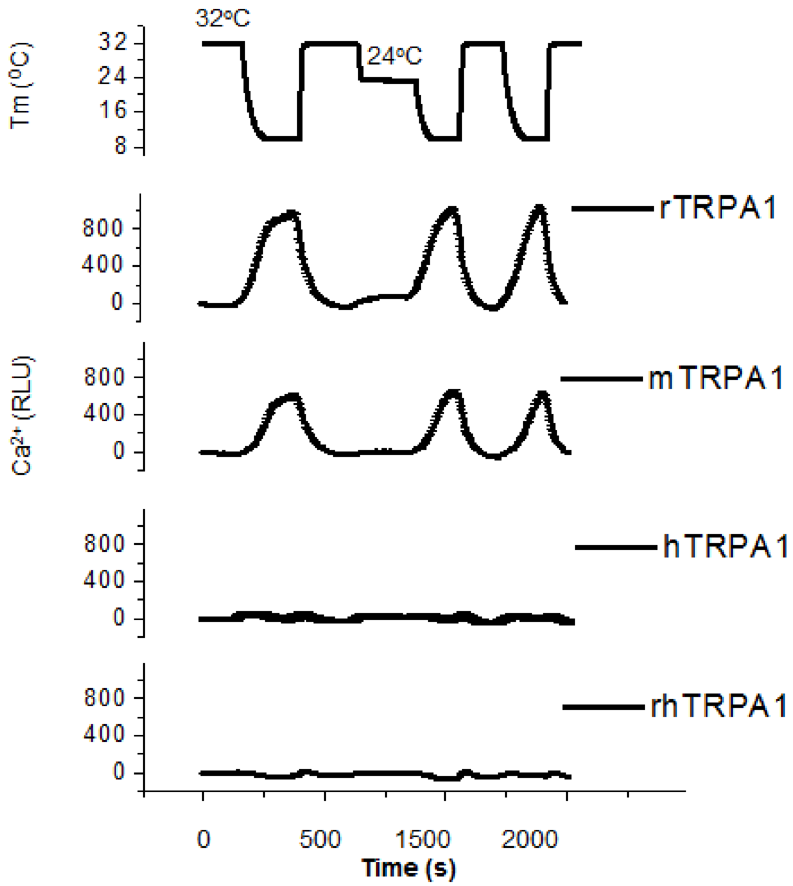

Supplementary Figure S2. Difference in cold responses between rodent and primate TRPA1. The cold-evoked Ca2+ responses in r- and mTRPA1 were reversible and independent of holding temperature (32 or 24°C). Cold did not elicit Ca^{2+} responses in h- and rhTRPA1. n = 12

Supplementary Figure S3

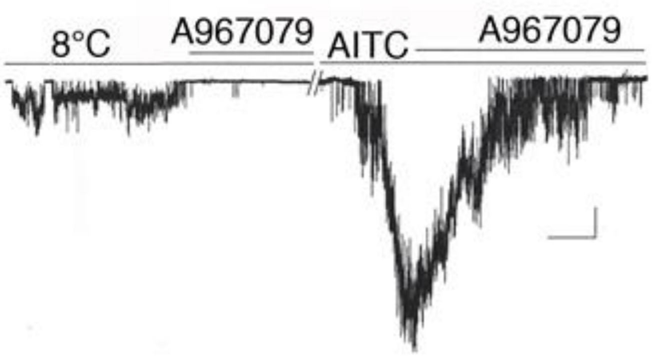

Supplementary Figure S3. rTRPA1 activities were blocked by 5 μM A967079. Cold- and AITC-evoked single channel activities were blocked by A967079 (- 60 mV), indicating TRPA1-specific conductance. The scale bars are 5 pA and 20 s respectively.

Supplementary Figure S4

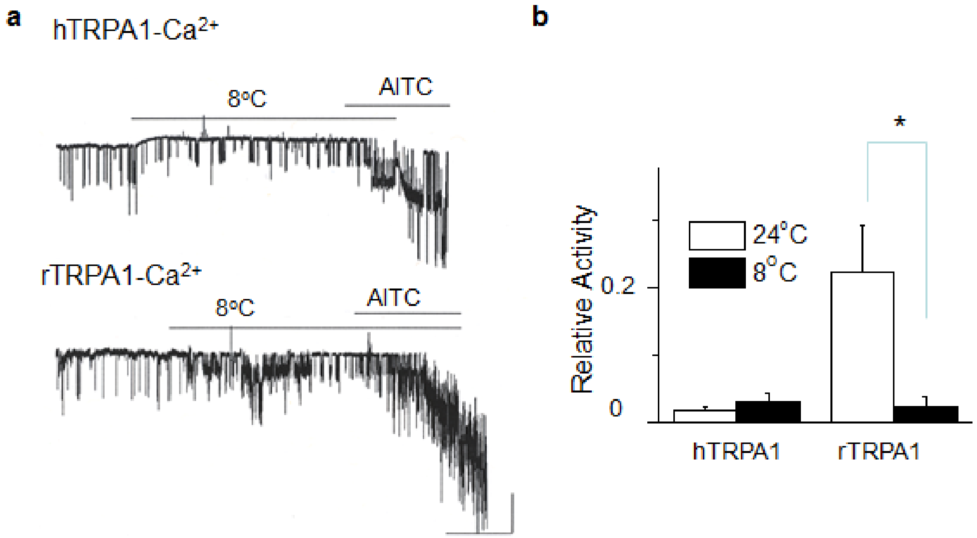

Supplementary Figure S4. Single channel activities of h- and rTRPA1 in 1 mM external Ca^{2+}. (**a**) Single channel traces at -60 mV. Scale bars are 5 pA and 10 s. (**b**) Relative activity of h- and rTRPA1 at 8°C and 24°C. Relative channel activity was determined by normalizing NPo against AITC response. Error bars are s.d. n = 4. * indicates p<0.05 from student's t-test.

Supplementary Figure S5

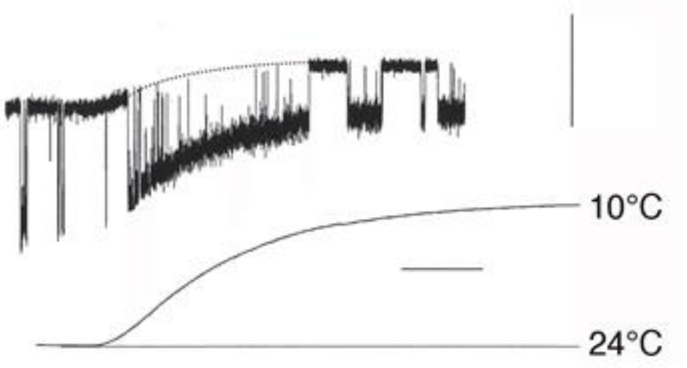

Supplementary Figure S5. Effects of temperature on rTRPA1 conductance. Cooling reduced unitary conductance in a progressive, but not a step-wise pattern. Scale bars are 5 pA and 2 s.

Supplementary Figure S6

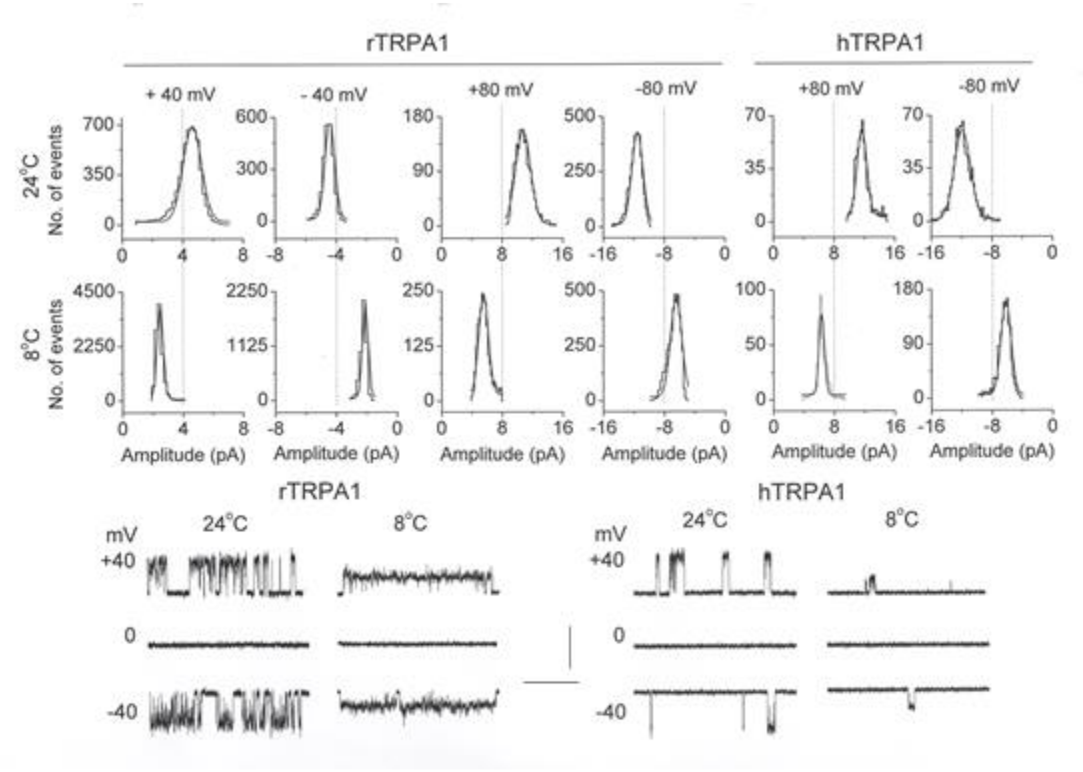

Supplementary Figure S6. Amplitude histograms of single channel openings of r- and hTRPA1 at 24 ^{o}C and 8 ^{o}C. A K^{+}-based perfusion and pipette solution was used, containing: 150 mM KCl, 1 mM $MgCl_2$, and 11 mM glucose. (PH7.3). Cell-attached patches were formed on HEK-293F cells expressing rTRPA1 or hTRPA1, and single channel openings were recorded at various membrane potentials. Representative amplitude histograms are shown. Current reversed at ~ 0 mV. At -40 mV, the single channel conductance for rTRPA1 were 71 pS and 21 pS at 24 ^{o}C and 8 ^{o}C, respectively. Scale bars are 3 pA and 100 ms. n = 4-5.

Supplementary Figure S7

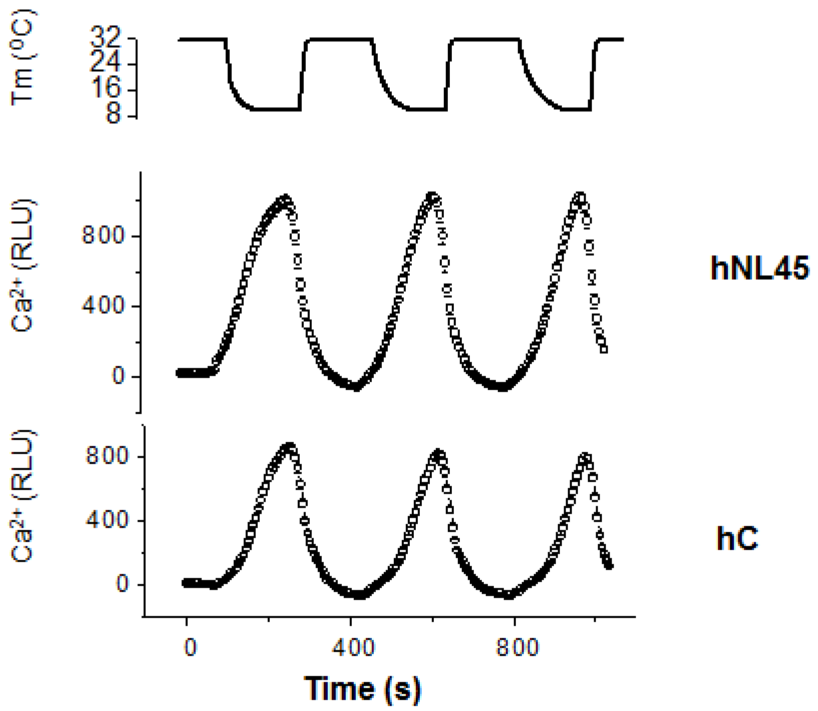

Supplementary Figure S7. On the rTRPA1 background, introducing N terminus through L45 linker [h(N-45)] or C-terminus of hTRPA1 (hC) retained cold activation. Fluorescence traces are shown as mean ± SD from 12 wells.

Supplementary Figure S8

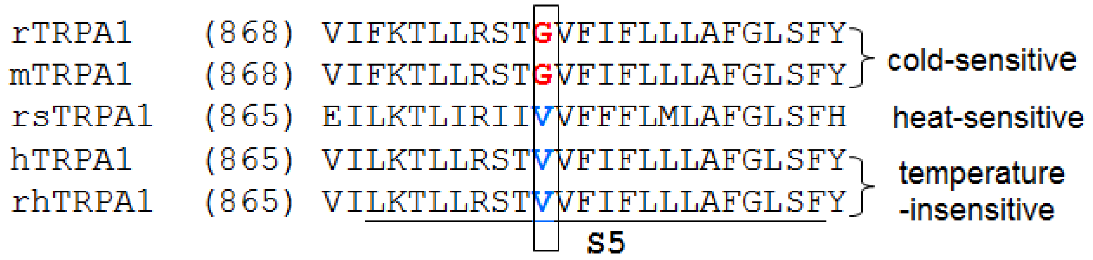

Supplementary Figure S8. Alignment of S5 domains. G878 are conserved in cold sensitive mTRPA1/rTRPA1; whereas V875 are conserved in heat-sensitive rsTRPA1 (rattlesnake) and temperature-insensitive hTRPA1/rhTRPA1.
